# Supplementary material for: Enhancer-driven 3D chromatin domain folding modulates transcription in human mammary tumor cells
Source: Life Sci Alliance. 2023 Nov 21;7(2):e202302154. doi: 10.26508/lsa.202302154 (PMC10663337; doi:10.26508/lsa.202302154)
Supplement: Supplementary file 1 [file LSA-2023-02154_TableS1.docx]

**Supplementary Table 1:** Fosmids used in this study.

| **Fosmid name** | **Whitehead (Sanger) name** | **Ensembl name** | **coordinates** | | **Size (bp)** | **midposition** |
| --- | --- | --- | --- | --- | --- | --- |
| Fos1 | *WI2-1069H1* | *G248P83181D1* | 100'857'188 | 100'893'758 | 36'570 (+) | 100'875'473 |
| Fos2 | *WI2-3198P11* | *G248P8001H6* | 100'930'139 | 100'972'364 | 42'226 (+) | 100'951'252 |
| Fos3 | *WI2-630J2* | *G248P80527E1* | 101'008'384 | 101'050'881 | 42'498 (+) | 101'029'663 |
| Fos4 | *WI2-2176P22* | *G248P87059H11* | 101'105'566 | 101'148'475 | 42'910 (+) | 101'127'021 |
| Fos5 | *WI2-758J11* | *G248P81257E6* | 101'238'076 | 101'275'616 | 37'541 (+) | 101'256'846 |
| PUM1 (3’) | *WI2-1091D18* | *G248P82995B9* | 31’359’975 | 31’403’626 | 43,652 (-) | 31’381’800 |
| PUM1 (5’) | *WI2-1757M9* | *G248P86664G5* | 31’540’133 | 31’581’406 | 41,274 (+) | 31’581’406 |
