# Supplementary material for: Enhancer-driven 3D chromatin domain folding modulates transcription in human mammary tumor cells
Source: Life Sci Alliance. 2023 Nov 21;7(2):e202302154. doi: 10.26508/lsa.202302154 (PMC10663337; doi:10.26508/lsa.202302154)
Supplement: Supplementary file 2 [file LSA-2023-02154_TableS2.docx]

**Supplementary Table 2:** Datasets used in this study.
